# Investigation an Antifungal Activity of Diclofenac Sodium against Hyphae Formation in Aspergillus Fumigatus with Attention to the Expression of Ef-1 Gene

**Published:** 2018-05

**Authors:** Sanaz NARGESI, Sassan REZAIE

**Affiliations:** Division of Molecular Biology, Dept. of Medical Mycology and Parasitology, School of Public Health, Tehran University of Medical Sciences, Tehran, Iran

## Dear Editor-in-Chief

*Aspergillus fumigatus*, a saprophytic airborne fungus, is mentioned as the most common invasive mold with mortality rates exceeds 50% in high-risk groups. Recently, with increases in the number of immunocompromised individuals, there has been a noticeable increase in the rate of invasive aspergillosis (1–3).

Translation elongation factors (EFs) are molecules for protein synthesis on ribosome. These molecules bind guanine nucleotides and in its GTP-liganded form can interact with aminoacyl tRNA to bring it to the A-site of the ribosome. Following hydrolysis of the GTP, EF-1, GDP is released from the ribosome. The activity and regulation of EF-1 *a* are expressed developmentally and that this regulation occurs at severa1 levels (4). Diclofenac sodium is a non-steroidal anti-inflammatory drug (NSAID) and is widely used for treatment of inflammatory disease (5). Additionally, anti-microbial effects of this drug have been documented in previous studies (6, 7). Inhibitory effects of this drug occurred by interfering in prostaglandin biosynthesis (8).

In the present study, antifungal activity of diclofenac sodium was investigated against hyphae formation in *A. fumigatus* with special attentions to the expression of EF-1gene.

To determine the minimum inhibitory concentration (MIC), the standard strain of *A. fumigatus* (ATCC 14489) was cultured 48 h at 35 °C in sabouraud dextrose agar medium. Inoculum suspensions were prepared by scraping the surface of colonies with a loop on sterile Tris-EDTA buffer with Tween 40 (0.05%). Spore suspension was then prepared at concentration of 5×10^4^ CFU ml^−1^ using hemocytometer method. Afterward, the suspension was treated with different concentrations of diclofenac sodium (25–900 μg/ml). Microplates (24 holes) were determined according to recommendations stated in the CLSI M38-A2 document (9). Fungal suspensions treated by diclofenac sodium alone (control positive) and untreated (Control negative) have also been considered. Finally, the cultured microplates incubated at 35 °C for 48 h. To investigate the level of EF-1 gene expression in *A. fumigatus*, a quantitative Real-time PCR was performed. Briefly, cultured *A. fumigatus* cells in the presence of 500, 700, 900 μg/ml of diclofenac sodium as well as treated (Control+) and untreated (Control−) fungal cells with diclofenac were considered. After incubation period, the harvested mycelia mass washed by phosphate buffer saline (PBS) 1X and grinded by liquid nitrogen to gain a fine mycelial powder. The obtained mycelial powder was used for RNA extraction by Guanidine Isothiocyanate method (GITC) (10). cDNA molecules were prepared by using random hexamer primers and reverse transcriptase enzyme, according to the defined protocol (RevertAid, Fermentase, Germany). Pairs of primer for amplification of EF-1 gene and β-actin gene (as housekeeping gene) were designed and synthesized (Sinaclone, Iran). Real-time PCR was carried out in order to comparison between levels of EF-1 mRNA as well as β-actin mRNA in treated and untreated cells. Operational program for amplification cycles was 95 °C 30 sec as initial denaturation, followed by 40 cycles of 95 °C for 10 sec, 60 °C for 30 sec. Real-time PCR system (StepOne-Plus TM, Applied Biosystems, USA) and Syber Green I dye (SYBR® Premix Ex Taq™ II) were used in this investigation.

**Fig. 1:**
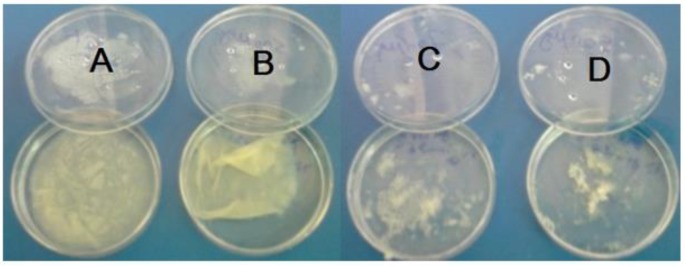
*Comparison the growth-level of* untreated *A. fumigatus* mycelial cells (A) with *A. fumigatus* mycelial cells treated with different concentrations of diclofenac sodium (B- 500 μg/ml, C-700 μg/ml, and D- 900 μg/ml)

By increasing of diclofenac sodium concentration, mycelium production have been decreased and deviation seen in their normal shape. Drug treatment in concentration of 500 μg/ml and more have indicated a significant inhibitory effect on the *A. fumigatus* growth (Fig. 1). In addition, measured levels of EF-1 mRNA in diclofenac treated and untreated cells revealed higher expression in untreated control cells (27.7) compared with fungal cells treated with 900 μg/ml of diclofenac (0.672) after normalization to β-actin (Table 1). Diclofenac can decrease the EF-1 gene expression. Diclofenac sodium, with a dose-dependent effect, can significantly reduce both; the production of mycelia and EF-1 gene expression in *A. fumigatus*.

**Table 1:** Comparison of EF-1 Gene expression in untreated control *A. fumigatus* mycelial cells (A) and *A. fumigatus* mycelial cells treated with 900 μg/ml diclofenac sodium (D)

***Gene***	***Type***	***Reaction Efficiency***	***Expression***	***Std. Error***	***95% C.I.***	***P(H1)***	***Result***
1	REF	0.695	1.000				
2	EF1 (A)	0.65	27.770	27.770 – 27.770	27.770 – 27.770	0.000	UP
4	EF1 (D)	0.57	0.672	0.672 – 0.672	0.672 – 0.672	0.000	DOWN

We suggest the performance of more clinical studies for validation possible usage of diclofenac sodium in order to treat aspergillosis infection.
